# Hematologic Adverse Effects of Prolonged Piperacillin-Tazobactam Use in Adults

**DOI:** 10.4274/tjh.2018.0127

**Published:** 2018-11-13

**Authors:** Aysun Benli, Serap Şimşek-Yavuz, Seniha Başaran, Atahan Çağatay, Halit Özsüt, Haluk Eraksoy

**Affiliations:** 1 Muş State Hospital, Clinic of Infectious Diseases and Clinical Microbiology, Muş, Turkey; 2 İstanbul University İstanbul Faculty of Medicine, Department of Infectious Diseases and Clinical Microbiology, İstanbul, Turkey

**Keywords:** Neutropenia, Leukopenia, Eosinophilia, Piperacillin-tazobactam, Adverse effects

## Abstract

**Objective:**

We aimed to find the incidence and risk factors of hematologic adverse effects of piperacillin-tazobactam (TZP).

**Materials and Methods:**

Adult patients who used TZP for more than 10 days were included in the study.

**Results:**

The incidence of leukopenia, neutropenia, and eosinophilia in 110 TZP therapy episodes was found to be 16.3%, 10%, and 10%, respectively. Lower Charlson Comorbidity Index score, lower initial leukocyte count, combination of TZP with another antibiotic, and total duration of TZP therapy were found to be independent risk factors for leukopenia, while initial higher eosinophil count (IHEC) and usage of TZP for >20 days were independent risk factors for neutropenia and IHEC and total duration of TZP therapy were independent risk factors for eosinophilia.

**Conclusion:**

Longer duration of therapy, combination with other antibiotics, younger age with fewer comorbidities, and IHEC could result in hematologic adverse effects in patients treated with TZP. Patients with IHEC may be more prone to allergic reactions, so immunological mechanisms may facilitate the development of hematological adverse effects of TZP.

## Abstract (tr)

**Amaç:**

Piperasilin-tazobaktamın (TZP) hematolojik istenmeyen etki insidansını ve risk faktörlerini bulmayı amaçladık.

**Gereç ve Yöntemler:**

On günden uzun süre TZP kullanan erişkin hastalar çalışmaya dahil edildi.

**Bulgular:**

Yüz on tedavi epizodunda lökopeni, nötropeni ve eozinofili insidansları sırasıyla %16,3, %10 ve %10 olarak bulundu. Charlson’ın Komorbidite İndeksi’nin düşük olması, başlangıç lökosit sayısının düşük olması, TZP ile başka antibiyotiğin kombine kullanılması ve TZP’nin toplam tedavi süresi; başlangıç eozinofil sayısının yüksek olması (BESYO) ve 20 günden uzun süreli TZP kullanımı; BESYO ve TZP’nin toplam tedavi süresi sırasıyla lökopeni, nötropeni ve eozinofili için bağımsız risk faktörleri olarak bulundu.

**Sonuç:**

TZP ile tedavi edilen hastaların tedavi süresinin uzun olması, kombine antibiyotik tedavisi almaları, daha genç yaşta daha az komorbiditelerinin olması ve BESYO hematolojik istenmeyen etkilerin gelişmesine neden olabilir. BESYO olması hastaların alerjik reaksiyonlara daha yatkın olmasına neden olabilir, bu nedenle immünolojik mekanizmalar TZP kullanımıyla hematolojik istenmeyen etkilerin gelişmesini kolaylaştırabilir.

## Introduction

Piperacillin-tazobactam (TZP) is a broad-spectrum semisynthetic antibiotic. It has increased activity against *Pseudomonas aeruginosa* when compared with other penicillins [1]. It is commonly used in nosocomial infections and many other conditions that require broad-spectrum antibiotics, such as febrile neutropenia. Adverse effects of TZP include hypersensitivity reactions and gastrointestinal, renal, and hematologic effects. Although the most frequently reported hematologic adverse effect of TZP is reversible neutropenia, Coombs-positive hemolytic anemia and thrombocytopenia are also reported [1,2]. After the observation of fever and neutropenia in some patients who received prolonged TZP therapy, we aimed to identify the incidence and risk factors for the development of these adverse effects.

## Materials and Methods

### Patient Selection

Adult patients (aged >18 years) who were given original TZP for more than 10 days at our faculty from January 2013 to December 2014 were included in the study. Usual adult doses were used and TZP was adjusted to renal function if necessary. Patients with HIV infection and hematologic malignancy, patients with leukopenia and neutropenia, and patients using systemic steroid therapy or chemotherapy within the last 3 months were excluded from the study. If the duration between two episodes of TZP therapy exceeded 1 month, those episodes were evaluated separately.

### Data Collection

Patient information was recorded on previously prepared forms by reviewing medical records. The Charlson Comorbidity Index (CCI) was calculated for all patients.

### Definitions

Leukopenia was defined as absolute leukocyte count of <4000 cells/mm^3^. Anemia was defined as hemoglobin level of <13.5 g/dL in males or <12 g/dL in females, or a decline of 2 g/dL in patients with low hemoglobin levels at the beginning of therapy. Thrombocytopenia was defined as absolute platelet count of <150,000 cells/mm^3^, neutropenia was defined as absolute neutrophil count of <2000 cells/mm^3^, eosinophilia was defined as absolute eosinophil count of ≥500 cells/mm^3^, and hypereosinophilia was defined as absolute eosinophil count of ≥1500 cells/mm^3^.

### Statistical Analysis

Statistical analyses were performed using SPSS 21 (IBM Corp., Armonk, NY, USA). The univariate analyses were investigated using chi-square tests, Fisher’s exact test, Student’s t-test, and Mann-Whitney U tests as appropriate. For multivariate analysis, the possible factors identified with univariate analyses were further entered into logistic regression analysis to determine independent risk factors for leukopenia, neutropenia, and eosinophilia. Hosmer-Lemeshow goodness-of-fit statistics were used to assess model fit and p<0.05 was considered statistically significant.

## Results

One hundred and ten TZP therapy episodes of 102 patients were included in the study. The epidemiological, clinical, and laboratory data of the patients are given in Table 1. Total TZP dose and duration of TZP therapy had no significant effect on the development of anemia or thrombocytopenia. However, they were detected as significant risk factors for the development of leukopenia (16.3%), neutropenia (10%), and eosinophilia (10%).

Drug fever appeared in five of the 11 neutropenic patients and in six of the 18 patients with leukopenia who were afebrile beforehand. All of the patients were alive until the end of TZP therapy. Therapy was continued with another antibiotic in 8 patients with leukopenia and in 5 patients with neutropenia. Body mass index was normal in all patients who developed leukopenia and neutropenia.

Characteristics of patients and statistical analysis with and without leukopenia, neutropenia, and eosinophilia during TZP therapy are given in Table 2. In multivariate analysis, lower CCI score, lower initial blood leukocyte count, combination of TZP with another antibiotic, and total duration of TZP therapy were found to be independent risk factors for leukopenia; initial higher blood eosinophil count (IHEC) and use of TZP for >20 days were found to be independent risk factors for neutropenia; and IHEC and total duration of TZP therapy were found to be independent risk factors for eosinophilia. The characteristics of leukopenia, neutropenia, and eosinophilia episodes are given separately in Tables 3, 4, and 5, respectively.

## Discussion

The incidence of leukopenia and neutropenia in patients treated with TZP for more than 10 days were found to be 16.3% and 10% respectively in our study. Incidence of neutropenia was found between 0.04% and 34% in previous studies [3,4,5]. The difference between neutropenia incidences may have resulted from the definitions of neutropenia, duration of TZP therapy, and study design. The total dose and duration of TZP therapy were also found to be the most frequently determined risk factors in the development of these adverse effects in previous studies [4,5,6]. The mechanisms and causes of TZP-induced leukopenia or neutropenia have not been clearly determined. It has been shown that TZP causes reversible proliferation arrest in myeloid cells with cumulative doses [7,8,9].

Duration of TZP therapy was detected as a significant risk factor for the development of leukopenia (21 days), neutropenia (19 days), and eosinophilia (13 days) in our study. Also in a study of 41 patients with bone-related infections, neutropenia developed in patients who used TZP for more than 18 days [4]. In another study that compared risks of neutropenia in patients treated with either TZP or ticarcillin-clavulanate, the risk of neutropenia was higher when children were treated with TZP than with ticarcillin-clavulanate and use of TZP for more than 2 weeks was found to be related to increased risk of neutropenia [5].

In some studies patients who developed neutropenia were found to be younger, as in our study [4,9]. However, these studies could not explain the mechanism behind this. We could find no other study identifying lower CCI as a risk factor for developing leukopenia or neutropenia during TZP therapy in adult patients. This situation could be explained by the role of immunological mechanisms in the hematologic adverse effects of TZP. Hypersensitivity responses against antimicrobial agents may be more effective in younger patients with better immune systems and no comorbid conditions. Additionally, we found IHEC as another independent risk factor for the development of neutropenia with TZP therapy. Patients with higher eosinophil counts were probably allergic to something previously and could be more prone to allergic reactions to antibiotics such as TZP as well; this could also be the reason for neutropenia and leukopenia. In another study, immunoglobulin G antibodies directed against penicillins and neutrophils were described and the authors concluded that an immune-mediated pathogenesis was highly probable in developing neutropenia with penicillin use [10].

Combination antibiotic therapy was found to be a risk factor for the development of leukopenia but not neutropenia in our study, and it was found as a risk factor also in developing neutropenia in another study [4]. Although the hematologic adverse effects of ciprofloxacin, which was the agent most frequently combined with TZP in our study, are mild and rarely seen[11], bone marrow suppression associated with ciprofloxacin use was shown. Combination antibiotic therapy with TZP should be limited to patients with severe life-threatening *Pseudomonas aeruginosa* infections and especially those with immunocompromising conditions because of the increased rate of adverse effects, including leukopenia, and the lack of evidence of either improved efficacy or decreased resistance [12].

### Study Limitations

Our study is novel in several ways: it includes the largest patient sample among studies on the same subject, we evaluated the hematologic adverse effects of TZP as a whole, and finally we analyzed the independent risk factors for development of leukopenia, neutropenia, and eosinophilia.

## Conclusion

It should be kept in mind that if TZP therapy is extended for more than 2-3 weeks, a patient could develop leukopenia, neutropenia, or eosinophilia, especially in cases of combination antibiotic therapy and in younger patients with fewer comorbidities. Although the consequences of TZP-induced hematologic adverse effects were not devastating, duration of hospital stay after the beginning of TZP was longer in patients with leukopenia and neutropenia. Therefore, younger patients with fewer comorbidities and patients with IHEC should particularly be monitored more frequently with complete blood counts. Although combination antibiotic therapy was not found as a risk factor for neutropenia, it was a risk factor for leukopenia and should be avoided unless necessary.

## Figures and Tables

**Table 1:**
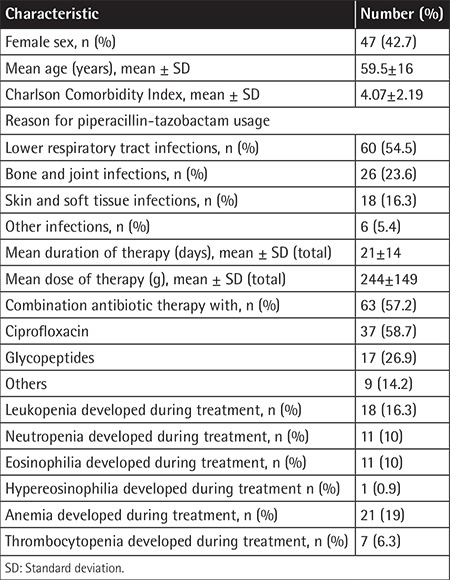
Characteristics of patients within 110 therapy episodes.

**Table 2:**
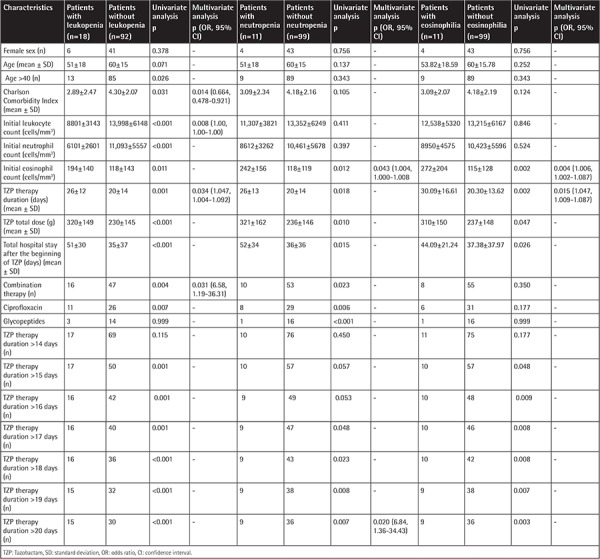
Characteristics of patients with and without leukopenia, neutropenia, and eosinophilia.

**Table 3:**
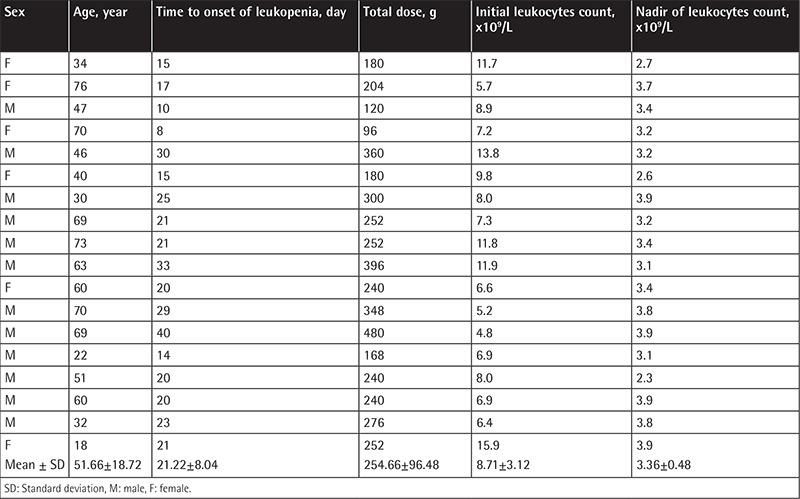
Characteristics of the 18 episodes of leukopenia.

**Table 4:**
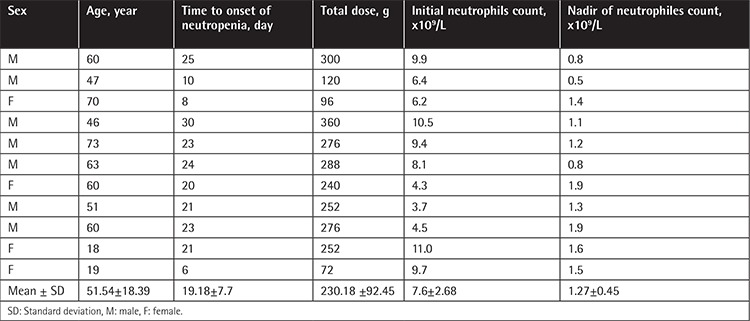
Characteristics of the 11 episodes of neutropenia.

**Table 5:**
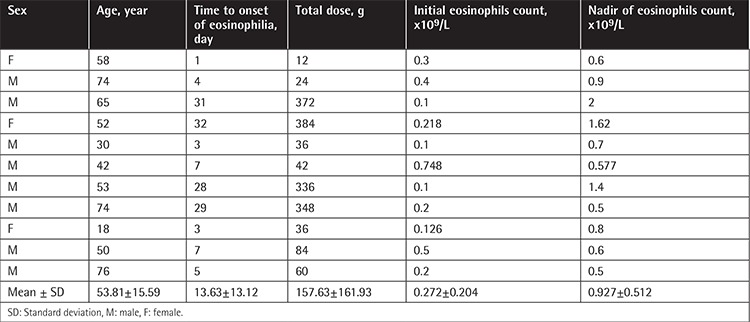
Characteristics of the 11 episodes of eosinophilia.

## References

[1] Doi Y, Chambers H (2015.). Penicillins and β-lactamase inhibitors. In: Bennett J, Dolin R, Blaser M, (eds). Mandell, Douglas, and Bennett’s Principles and Practice of Infectious Disease. Philadelphia, Elsevier Saunders.

[2] Daley D, Mulgrave L, Munro S, Smith H, Dimech W (1996). An evaluation of the in vitro activity of piperacillin/tazobactam. Pathology.

[3] Scheetz MH, McKoy JM, Parada JP, Djulbegovic B, Raisch DW, Yarnold PR, Zagory J, Trifilio S, Jakiche R, Palella F, Kahn A, Chandler K, Bennett CL (2007). Systematic review of piperacillin-induced neutropenia. Drug Saf.

[4] Peralta FG, Sanchez MB, Roiz MP, Pena MA, Tejero MA, Arjona R (2003). Incidence of neutropenia during treatment of bone-related infections with piperacillintazobactam. Clin Infect Dis.

[5] Lemieux P, Gregoire JP, Thibeault R, Bergeron L (2015). Higher risk of neutropenia associated with piperacillin-tazobactam compared with ticarcillinclavulanate in children. Clin Infect Dis.

[6] Reichardt P, Handrick W, Linke A, Schille R, Kiess W (1999). Leukocytopenia, thrombocytopenia and fever related to piperacillin/tazobactam treatment - a retrospective analysis in 38 children with cystic fibrosis. Infection.

[7] Kumar A, Choudhuri G, Aggarwal R (2003). Piperacillin induced bone marrow suppression: a case report. BMC Clin Pharmacol.

[8] Khan F (2005). Severe neutropenia secondary to piperacillin/tazobactam therapy. Indian J Pharmacol.

[9] Ruiz-Irastorza G, Barreiro G, Aguirre C (1996). Reversible bone marrow depression by high-dose piperacillin/tazobactam. Br J Haematol.

[10] Neftel KA, Wälti M, Spengler H, von Felten A, Weitzman SA, Bürgi H, de Weck AL (1981). Neutropenia after penicillins: toxic or immune-mediated?. Klin Wochenschr.

[11] Arcieri GM, Becker N, Esposito B, Griffith E, Heyd A, Neumann C, O’Brien B, Schacht P (1989). Safety of intravenous ciprofloxacin. A review. Am J Med.

[12] Vardakas KZ, Tansarli GS, Bliziotis IA, Falagas ME (2013). β-Lactam plus aminoglycoside or fluoroquinolone combination versus β-lactam monotherapy for Pseudomonas aeruginosa infections: a meta-analysis. Int J Antimicrob Agents.

